# Postthyroidectomy Horner's Syndrome

**DOI:** 10.1155/2012/316984

**Published:** 2012-10-21

**Authors:** Ramon Vilallonga, José Manuel Fort, Alejandro Mazarro, Oscar Gonzalez, Enric Caubet, Giancarlo Romero, Manel Armengol

**Affiliations:** ^1^Endocrine, Metabolic and Bariatric Unit, General Surgery Department, Vall d'Hebron Universitary Hospital and Center of Excellence for the EAC-BC, Passeig de la Vall d'Hebron 119-129, 08035 Barcelona, Spain; ^2^General Surgery Department, Vall d'Hebron Universitary Hospital, Passeig de la Vall d'Hebron 119-129, 08035 Barcelona, Spain

## Abstract

Horner's syndrome (HSd) results from an injury along the cervical sympathetic chain, producing ipsilateral miosis, ptosis, enophthalmos, and facial anhydrosis. Although more commonly associated to malignant tumors affecting the preganglionar segment of the sympathetic chain (especially in the lung apex), HSd has been described as a rare complication of thyroid surgery. We herein report a case of HSd after completing total thyroidectomy.

## 1. Introduction

Described by Bernard (1853) and Horner (1869), Horner's syndrome (HSd) consists of a tetrad defined by unilateral miosis, ptosis, enophtalmos, and facial anhydrosis. It results from an injury along the ipsilateral cervical sympathetic chain, usually in the preganglionic portion. The most frequent cause of HSd is neoplasms, being the malignant ones more common than the benign ones. However, when secondary to thyroid pathology, and against the general belief, HSd is more often due to benign thyroid pathology [1].

## 2. Case Presentation

**Figure 1:**
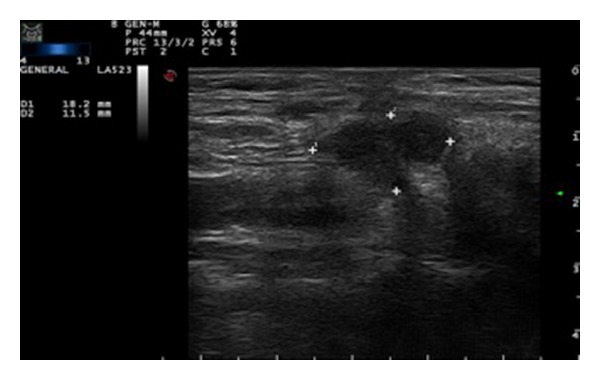
Hypoechogenic and avascular mass compatible with thyroidal remains.

**Figure 2:**
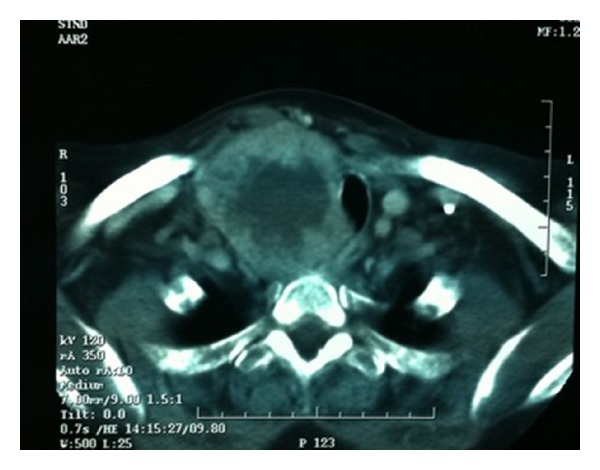
CT scan showing the mass pushing the trachea and occluding its lumen.

A 79-year-old woman presented with a right cervical mass. She had undergone a subtotal thyroidectomy in 1986 for multinodular goiter, and the results of the histopathological studies revealed no malignancy. Twenty-four years after surgery, the patient complained about mass, pain, and swallowing difficulty. The only biochemical remaining sequela was subclinical hypothyroidism. Imaging studies that were performed (ultrasound—Figure 1—and CT scan—Figure 2) revealed an enlarged right thyroid lobe and a right paratracheal mass displacing (but not invading) the carotid artery, the esophagus, and the trachea. A fine needle aspiration cytology (FNAC) was performed but no conclusion could be given according to the cytology found.

Surgery was performed, finding a thyroid gland strongly adhered to the carotid sheath and invading the posterolateral side of the esophagus. The recurrent laryngeal nerve was identified and respected. During the surgery, the patient suffered from bradycardia secondary to carotid manipulation, being reverted with atropine.

The first postoperative day, the physical exploration revealed right ptosis and miosis, being diagnosed with Horner's syndrome. Hematoma and seroma were ruled out as a cause after a careful exploration, and HSd was attributed to damage to the communication between the cervical sympathetic chain and the recurrent laryngeal nerve, associated to palsy of the right vocal cord (Figure 2). Final histopathological analysis revealed poorly differentiated insular carcinoma of the thyroid, an infrequent finding usually related to a low survival rate (overall five-year survival rate near 20%). The patient was discharged from the hospital and followed a radioactive iodine protocol, without further complications during the followup. To this date (twenty-four months after surgery), she has not complained from additional cervical masses suggestive of recurrence, although the formerly described ptosis and miosis still remain. A stroboscopy has been performed and shows a palsy of the right vocal chord in median position with mild edema and no signs of atrophy. During closure of the glottis, dynamic exploration revealed hyperkinesia of the left hemilarynx.

## 3. Discussion

In our report, the malignancy was confirmed finally. Major cause of Horner's syndrome remains surgery, with malignant lesions being twice as frequent as benign tumors [2]. Some authors concluded that Horner's syndrome was more often due to benign thyroid diseases than to thyroid malignancies. However, in our case, although initially the patient had a recurrent goiter, the final histopathology confirmed a poorly differentiated insular carcinoma.

We suspect that the lesion was produced by the local trauma to the sympathetic chain during retraction of the carotid sheath (the patient did a bradycardia during the surgery). Other possible causes of damage are the anatomical variations, a postoperative hematoma compressing the sympathetic chain or even an ischemic damage to the sympathetic chain.

Surgeons have to be aware of this complication when performing thyroid surgery in complicated cases. We understand by complicated cases tumour with a late presentation, cancer, size > 10 cm, imaging appearance of proximity to adjacent structures, airway stenosis, and so forth. 

Although HSd secondary to compression by a thyroid mass can improve after surgery, the surgeon must be aware that virtually any cervical surgery, and especially thyroid surgery, may be a source of iatrogenic Horner's syndrome. In a series of 495 thyroidectomies, Cozzaglio et al. described one case (0, 2%) of postsurgical Horner's syndrome [2].

Multiple injury mechanisms have been described to cause HSd after a thyroidectomy, and surgeons devoted to thyroid gland surgery must know them in order to avoid and treat them promptly if needed. The most common causes of postthyroidectomy HSd are haematoma compressing the cervical sympathetic chain, ischemic neural damage, stretching of neural structures, and disruption of the connection between the sympathetic chain and the recurrent laryngeal nerve [3, 4].

## 4. Conclusion

Horner's syndrome has been described as a rare complication of thyroid surgery, affecting, up to 0, 2% of patients submitted to thyroidectomy. It is essential to the surgeon to be able to promptly diagnose it during the immediate postoperative period, as it may be secondary to reversible causes as seroma or hematoma. Considering the incidence of thyroid pathology, we would like to emphasize the importance of being aware of this rather rare complication, both as a pre- and postsurgical finding.
